# Expanding the role of metabolism in whole plant models

**DOI:** 10.1038/s41540-026-00773-5

**Published:** 2026-07-04

**Authors:** Karuthedath Shaijal, Meera Joshi, Sanu Shameer

**Affiliations:** School of Biology, Indian Institute of Science Education and Research Thiruvananthapuram, Thiruvananthapuram, India

**Keywords:** Computational biology and bioinformatics, Plant sciences, Systems biology

## Abstract

Whole-plant models capture the dynamics of plant architecture and physiology. However, the representation of metabolism in these frameworks remains limited. This work surveys the representation of metabolism in popular mechanistic whole-plant modelling frameworks. We next discuss the scope of using constraint-based metabolic models to study metabolism at the whole-plant scale and finally present a case for integrating metabolic models with other models to overcome the limitations of individual models.

## Introduction

Understanding plant growth is central to developing strategies to meet the rising global demands for food and agricultural productivity^1^. Plant growth is an integrated outcome of metabolic processes that convert assimilated Carbon (C), Nitrogen (N), Phosphorus (P), Sulphur (S) and other plant nutrients into biomass using light energy. Plant growth can be studied at different spatial scales, ranging from cell-level processes like cell division and differentiation^2,3^ to plant-level processes like the appearance of new organs and expansion of existing ones^4^, all the way up to landscape-level processes such as crop yield or vegetation per hectare^5,6^. Analysing plant-level and ecosystem-level growth, in particular, is essential to develop successful strategies to future-proof global agricultural productivity. An adaptation of the Monteith equation^7^ identified conversion efficiency, the efficiency at which light energy is converted into organic form, as the most rate-limiting step in the conversion of energy from the sun into plant yield. Another study revealed sites for accelerating growth and yield in land plants^8^ and motivated engineering efforts to improve photosynthesis and crop yield in the post-green revolution era^9,10^. In addition to plant-centric studies, empirical studies that forecast agricultural yield also demonstrate that including weather variables such as temperature and precipitation indices substantially improves plant growth predictions^11^. Together, these studies hint at the multiscale complex nature of plant growth, in which resource capture, metabolic conversion efficiency, biomass allocation and environmental variability jointly determine plant performance. While empirical analysis and physiological measurements have been instrumental in identifying the key drivers of growth and yield, translating these insights into predictions facilitating crop management efforts, particularly under increasingly unpredictable and volatile growing conditions, requires mathematical modelling frameworks that can integrate the above-mentioned processes across organs, tissues and developmental time^12^.

**Fig. 1:**
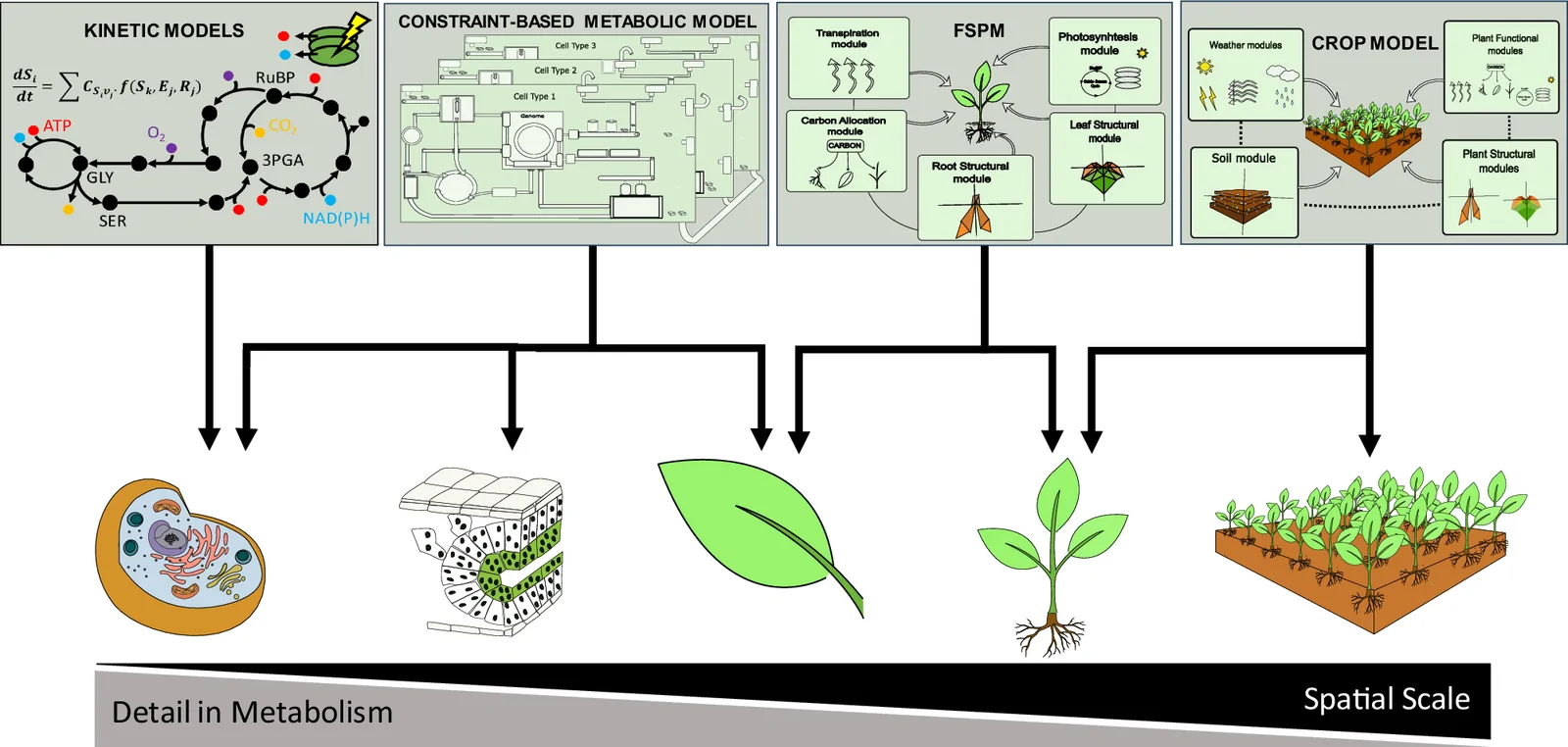
Trade-off between spatial scale and metabolic detail in mechanistic plant models. Different modelling approaches have been applied to model processes at various spatial scales. Kinetic models, the most detailed of all metabolic models, are popularly used to model growth in small systems. Constraint-based models, which do not account for enzyme kinetics, are capable of modelling growth in tissue, multitissue and organ-level models. This modelling approach has also been used to model growth at the whole plant level, but only in “big-leaf” models. Function-Structure Plant Models (FSPMs), which show limited representation of metabolism but are able to account for microclimate and canopy effects, are popularly used to model growth at the organ and plant levels. Crop models which rely on more empirical relationships to estimate growth are popularly used to model plant growth at the field scale.

Mechanistic models describe biological systems, be it a single cell, tissue, organ, whole plant or an entire community, using mathematical equations that explicitly represent the underlying biological processes^13^. By formalising our understanding and hypotheses of plant behaviour, such models offer conceptual clarity, facilitate mechanistic understanding and reveal knowledge gaps^14^. Multiscale mechanistic models offer a means to integrate biological processes operating at different spatial and temporal scales into a single framework^15^. At cellular and tissue levels, ordinary differential equations (ODEs) based models can be used to describe non-linear dynamics of the system based on rate laws and enzyme kinetic parameters. Such models have been used to analyse photosynthesis and primary C metabolism in great detail^16,17^. The relatively simple Farquhar-von Caemmerer-Berry model (FvCB), which describes C_3_^18^ photosynthesis as two processes, is one of the most popular models for describing photosynthesis mechanistically. Approaches at similar scales include partial differential equation-based signal transduction models^19^ and Boolean models of gene regulation^20^ and signal transduction^21^. These models, however, rely on difficult-to-gather context-specific information such as kinetic parameters, metabolite concentrations, enzyme concentrations, transcriptomics data, complex mathematical relationships, and hence often have limited coverage of metabolism. Cellular metabolism can alternatively be studied using constraint-based modelling of genome-scale metabolic models (GSMMs), which account for all metabolic capabilities encoded in the genome via gene-protein reaction associations. These models rely only on constraints on nutrient uptake and internal metabolic fluxes and have been applied to a wide range of steady-state plant systems^22–25^. At higher spatial scales, plant growth simulations can be driven by core physiological processes using what is commonly referred to as process-based models or functional plant models (FPMs)^13^. These models integrate mechanistic modules describing different contributors to growth, such as photosynthesis^18^, plant hydraulics^26^ and photoassimilate translocation^27^ to predict plant growth under a given set of environmental conditions. Overall, we can see that as the scale at which plant growth is studied increases, the level of detail in metabolism described decreases [Fig. 1].

The reduction in metabolic detail at higher scales can significantly limit the mechanistic interpretability of plant growth and development. Detailed representation of metabolic processes provides insight into the emergent physiological behaviour, including source-sink interactions, biomass allocations and stress response. The integration of metabolic processes also enables the identification of biochemical bottlenecks and candidate targets for genetic manipulation. For example, an enzyme-constrained metabolic model incorporating temperature-dependence of enzyme activity has been shown to identify genes involved in temperature-resilient growth of *Arabidopsis thaliana*^28^, accelerating efforts to develop stress-resistant crop varieties. Similarly, a constraint-based model of tomato leaf metabolism predicted that switching glutamate synthetase (GS) from chloroplast to the mitochondria could enhance leaf metabolic efficiency, a prediction that was experimentally validated in transgenic plants^29^. Dynamic ODE-based models of photosynthesis have also predicted enzymatic targets for improving carbon assimilation, including sedoheptulose-1,7-bisphosphate and fructose-1,6-bisphosphate aldolase^30^, which was later shown to enhance yield in transgenic tobacco^31^.

Plant growth is, however, shaped not only by physiological functions but also by plant architecture^32–35^. Functional-Structural Plant Models (FSPM) are developed to bridge the gap between descriptive structural models and process-based crop models^36^. Functional Structural Plant Modelling (FSPM) can simultaneously depict both the dynamics in plant architecture and physiology during its life-cycle^37^. One of the earliest examples of FSPM is the model proposed for the root architecture development based on the inter-organ competition for the photoassimilates in phloem^38^. Since then, FSPMs have been extensively used in multiple plant systems, including rice^39^, cut-rose^40^, rapeseed^41^, and barley^42^. These environment-sensitive models offer organ-level and individual-level representation of plant processes, making them well suited to study the contribution of plant architecture, light interception, photosynthate allocation and resource remobilisation towards plant growth. These characteristics facilitate the application of these models in precision breeding and crop ideotype design^43^. A defining feature of FSPMs is the feedback between its structural and functional components. Environmental conditions will regulate functions (photosynthesis, C allocation, etc.), which will result in changes to 3D structure (growth, canopy arrangement, leaf area, etc.), thereby influencing subsequent plant functions^37^. The mechanistic nature of FSPMs makes it possible to scale them up from plant-level to plant population and community levels. For example, an FSPM generated by combining the structural model ‘ADEL-Wheat’ with a functional model ‘Nested radiosity’ (stimulates the light interception by the canopy) has been used to study the effect of red:far-red ratio when accounting for light scattering in the emergence of tiller buds in wheat populations^44^. Similarly, phenotypic responses to the photosynthetically active radiation in cucumber under shaded canopy have also been studied using FSPMs^45^.

At larger scales, real-world weather, soil, water availability and other environmental parameters are combined with structural and functional modelling to form Crop Simulation Models (CSM)^46^. In these models, environmental factors, soil processes, water availability, plant physiology and structural growth are represented as interacting modules that together predict the dynamics at the crop level, instead of individual plant models. WOrld FOod STudies (WOFOST)^47^, Decision Support System for Agrotechnology Transfer (DSSAT)^48^, Bioenergy Crop models (BioCro II)^49^ and Agriculture Production System Simulator (APSIM)^50^ are a few of the major general crop models. These are customised to specific crops for species-specific crop models^51–60^.

Mechanistic modelling frameworks are capable of describing plant processes to great detail^61^; however, many of these models are purpose-built and can be plant- or scenario-specific^15^. For example, the Arabidopsis framework model, which combines an FSPM with a C dynamic metabolic model, a circadian clock model, a photoperiodism model, a light signalling model and a phenology model, is capable of simulating plant growth and development in wild-type and clock mutant Arabidopsis plants^62^. The same model, however, cannot be used to study growth in other plant species or even other Arabidopsis mutant plants. Generic plant modelling frameworks such as GREENLAB^63^, BioCro^49^ and WOFOST^47^ offer powerful alternatives in the form of modular components that describe plant processes that can be customised based on the plant system and environmental conditions.

As described earlier, understanding systems operating at multiple scales could be challenging; however, initiatives like Crops In Silico are focused on developing generalised multi-scale modelling frameworks to integrate gene regulation, signalling, metabolism, and plant development to large whole plant or ecosystem scale^64^. Examples of such implementation are evident in many FSPMs. For example, in barley, a signal transduction network model of gibberellic acid was coupled with a light response model and a structural model^42^ to account for the effect of gibberellic acid signalling and shade avoidance on plant growth. To date, many powerful, purpose-built and generalised frameworks to describe plant growth have been developed. Some of them will be discussed in detail in the next section. Recognising the value in combining existing resources, tools to enable cross-platform and cross programming language integration of plant models have also been developed by the plant modelling community^65^. In this review, we discuss the representation of metabolism in existing whole plant models, the integration of constraint-based models into whole plant models to enhance this representation, the challenges in the integration process and future prospects

## Survey of functional plant models, functional structural plant models and crop models

As described earlier, multiple purpose-built FPMs, FSPMs, and crop models have been developed in recent years. While these frameworks differ substantially in their computational architecture, biological scope and structural complexity, they also vary considerably in the extent to which metabolism is mechanistically represented. This section primarily surveys this with a particular focus on how photosynthesis, carbon allocation, respiration, nutrient assimilation and dynamics in metabolite levels are incorporated. The information is also summarised in Table 1.

**Table 1:** Table summarizing the level of metabolism and environmental sensitivity in the surveyed models

Model	Photosynthesis - Level	Nutrients			Environmental Sensitivity				
		Carbon	Nitrogen	Specific Metabolite	Temperature	Light	Wind	Water	Soil
**AmapSim**^66^	–	–	–	–	–	–	–		
**ARCHIMED**^67,68^	✓	✓	–	–	✓				
**RATP**^69^	✓✓	✓	–	–	✓	–	✓	✓	-
**GroIM**P^72^	✓✓	–	–	–	–	–	–	–	-
**3dCAP**^84^	✓✓	–	✓	–	✓	✓	–	–	-
**GREENLAB**^63^	–	–	✓	–	✓	–	–	✓	-
**ALFALFA**^90^	✓✓	–	–	–	–	–	–	–	-
**Cropbox-Garlic**^93^	✓✓	✓	–	–	✓	✓	–	–	-
**GOSSYM** Cotton^95^	✓✓	✓	✓	–	✓	✓	✓	✓	-
**ADEL-Maize**^100^	–	✓	–	–	✓	✓	–	–	-
**L-egume**^101^	–	✓	✓	–	✓	✓	–	✓	✓
**L-Cucumber**^45^	✓✓	✓	-	–	✓	✓	–	–	-
**Arabidopsis Framework v2**^62^	✓✓	✓	-	Malate + Fumarate added	✓	–	✓		
**V-Mango**^109^	✓	✓	–	Organic compounds (sucrose, fructose, glucose, citric and malic acid)	✓	✓	–	✓	-
**CN-Wheat**^111^	✓✓	✓	✓	Nitrate, triose phosphates, sucrose, amino acids, starch	✓	✓	✓	–	✓

The checkmarks represent the level of detail; the higher the number of checkmarks, the greater the detail. Checkmarks for environmental sensitivity indicate whether it exists.

### Models with minimal or no explicit representation of metabolism

Several structural and architectural plant models focus primarily on describing plant morphology and developmental dynamics without explicitly representing metabolism. These models are designed to simulate organogenesis, branching patterns or canopy architecture, and therefore rely largely on developmental rules. An example of such a modelling framework is **AmapSim**^66^, a cellular automaton-based model capable of simulating architectural developments during plant growth in a wide variety of plants, ranging from *Pinus halepensis* trees to *Arabidopsis Thaliana* rosettes. This framework simulates the plant’s 3D structure based on the concept of basic growing units called ‘virtual buds’. Growth of each bud is directed by its physiological age and the reference axis, and growth is executed by a semi-Markovian process. The AmapSim model is included with the AMAPStudio modelling software and can be obtained by contacting the AMAPStudio team (https://amapstudio.cirad.fr/contact).

### Models with a simplified empirical representation of metabolism

Most existing whole-plant models represent metabolism indirectly through empirical or semi-mechanistic descriptions of photosynthesis, respiration and biomass accumulation. In these models, carbon assimilation is commonly simulated using the FvCB photosynthesis model and its extensions, while downstream metabolism is abstracted into source–sink relationships or biomass partitioning rules. Some of the popular plant models that fall into this category are described below.

**ARCHIMED**^67,68^ is a 3D virtual plant modelling platform that enables the simulation of biophysical processes associated with plant growth. The model is implemented as a standalone application on the Java-based AMAP studio (https://amapstudio.cirad.fr/soft/archimed/start). Canopy radiation, leaf energy balance, leaf temperature and sap flow within the architecture are calculated while accounting for feedback between the individual mechanisms. Calculated photosynthetically active radiation availability, stomatal conductance and leaf temperature are used to simulate CO_2_ assimilation using the FvCB model. Recently, the ARCHMED modelling platform has been developed in two modules: (a) the publicly available ARCHIMED-φ, which simulates the aforementioned biophysical processes and (b) the yet to be released ARCHMIED-γ, which simulates plant development, growth and yield (https://archimed-platform.github.io/).

**RATP**^69^ stands for Radiation, Absorption, Transpiration and Photosynthesis. RATP is capable of using voxels of different leaf densities to describe the plant canopy in several vegetation types. While the model is unable to simulate changes to plant architecture, it can compute radiation transfer, transpiration and photosynthesis of static 3D canopies given the microclimate within the canopy and leaf physiological and structural properties. The model uses a radiative transfer model^70^ combined with an energy balance model to compute incident radiation, and photosynthesis is modelled based on the FvCB model. Fortran implementations of RATP version 2 can be accessed via Python using the OpenAlea platform^71^(https://pyratp.readthedocs.io/en/latest/).

**GroIMP** (Growth grammar related Interactive Modelling Platform) is an open-source Java-based modelling platform that enables modelling and visualisation of plant growth using rule-based modelling^72^. The models are written in eXtended L-systems(XL), a Java extension to specify L-systems models^73^ using relational growth grammars. GroIMP has been used to simulate plant growth in many model plant species^74–77^. GroIMP has been used to study the optimisation of spatial light distribution for tomatoes in greenhouses^78^. Functionally, the GroIMP Tomato model can simulate photosynthesis based on the FvCB model, and photosynthetically active radiation (PAR) in the model is simulated using a nested radiosity model^79^. A GroIMP-based *Brassica nigra* model has also been used to simulate plant-plant and herbivore-plant interactions^75^. This GroIMP model uses the Monte Carlo path tracing to calculate incident PAR, and a light response curve is used to estimate C assimilation per leaf. The model also has empirical representations for dynamic C allocation and growth-associated respiration based on sink strength and shade avoidance. In the GrapevineXL^77^ model, the GroIMP framework was used to simulate both above and below-ground systems of grapevines. GrapevineXL has an extended FvCB module representing photosynthesis in detail and stomatal conductance, leaf temperature and transpiration using empirical relationships^80^. The model also has an empirical Absicic Acid (ABA) sensitive model to represent plant hydraulics^81^. A GroIMP Cut-rose^40^ FSPM has been shown to be capable of representing a bent shoot canopy. Incident light is the model used, which is calculated based on^82^ with the Monte Carlo ray-tracer, and leaf photosynthesis is calculated according to the FvCB model. The model also uses a bud-break model to account for plant pruning events. In the case of a GroIMP-based soybean^74^ model, turgor-pressure-driven growth has also been shown to be capable of simulating plant growth. Structural components were described using a modified linear phytomer model^74^, which simulates the above-ground plant architecture. The GroIMP Soybean model also accounted for photosynthesis, stomatal conductance and transpiration using a coupled P-SC-T model^83^, and uses sugars as a metabolite to control osmotic potential and thereby cell expansion. The latest version of the GroIMP framework and instructions to use it have been made available at https://grogra.de/.

**3dCAP**^84^ is a 3D static model of rice canopy architecture developed to study the effects of structural traits on radiation use efficiency. The model is parameterised based on field-grown rice and is capable of calculating light interception and distribution using a forward ray-tracing algorithm and a mechanical model for weight distribution. Radiation use efficiency is calculated in the model using the semi-empirical FvCB model. 3dCAP is implemented on the MATLAB engine, and executable files are available at https://rootchang.github.io/3dCAP/.

**GREENLAB**^63^ is a dynamic semi-mechanistic 3D modelling framework capable of simulating and integrating plant organogenesis, structure and functions. The modelling framework applies a dual-scale automaton to simulate organogenesis based on deterministic architectural rules. The model uses Beer-Lambert’s law to estimate light interception and calculates biomass assimilation rate empirically. Biomass allocation in GREENLAB models is dynamic and calculated based on the sink strength over development. The flexible nature of the GREENLAB model makes it possible for it to be calibrated for multiple plant species, including Teak^85^, coffee^86^, rapeseed^87^, tomato^88^, and maize^89^. GREENLAB and its various implementations are available on MATLAB, Scilab, Java or C + + from https://greenlab.cirad.fr/GLUVED/html/P3_Tools/Tool_simul_003.html.

**ALFALFA**^90^ is a model capable of modelling organogenesis, structural dynamics and biomass production in *Medicago sativa* plants. In this model, plants are described in Markov states and Markov chains based on thermal time are used to describe organogenesis and transition from one Markov state to the next. Along with the simulation of shoots using phytomer units and modified phytomer units (cotyledons, leaves, petiole and inflorescences), the model captures root development using Basic Unit Roots (BURs) of taproot, first lateral root and second lateral root. The model accounts for self-shading in the canopy, and models photosynthesis using a negative exponential descending model. The model uses a Gaussian integral method^91^ to calculate average photosynthetic rate and biomass distribution to organs, following the GreenLab method^92^. ALFALFA 1.4 is available for download from the University of California Agriculture and Natural Resources website https://ipm.ucanr.edu/IPMPROJECT/alfalfa.html.

**Cropbox-Garlic**^93^ is a mechanistic C + + model capable of describing the phenology and morphology of Hardneck Garlic (*Allium sativum*). The model focuses on incorporating the nonlinear effect of temperature and bulb storage on leaf development and morphology during plant growth in Hardneck Garlic at a crop level. The detailed structural component of the model permits it to accurately estimate light interception under dynamic growing conditions. Photosynthesis in the model is captured by a modified FvCB model coupled to the BWB stomatal conductance model^83^. The model also accounts for growth and maintenance respiration and leaf senescence. Plant growth observations are used to estimate dynamic photosassimilate partitioning coefficients in the model. An updated version of the Cropbox-Garlic model has been shown to connect functional modules to predict garlic yield^94^. The model and instructions on how to use it can be found in a GitHub repository (https://github.com/uwkimlab/cropbox-garlic).

**GOSSYM** Cotton^95^ model combines the cotton-specific physiology-based crop level model Gossypium Simulation (GOSSYM)^96^ with 3D structural modelling to simulate a virtual cotton plant. The GOSSYM model uses an empirical relationship between incident light intensity and gross photosynthesis to calculate the gross photosynthetic rate of the plant canopy. Maintenance and growth respiration is subtracted from the gross photosynthesis to estimate net photosynthesis, which is then distributed among the organs^97^. In recent versions of GOSSYM^98^, the empirical calculation of photosynthesis has been replaced by a mechanistic approach involving the FvCB photosynthesis model coupled to a BWB stomatal conductance^99^ and a leaf energy balance model^83^. The latest version of the GOSSYM Cotton model can be found in a GitHub repository (https://github.com/USDA-ARS-ACSL/GOSSYM-2DSOIL).

**ADEL-Maize**^100^, standing for Architectural model of DEvelopment based on L-systems Maize, is a maize crop simulation model (CSM) that is capable of describing the 3D model of maize crop from an organ level, building up to the canopy level. The model uses a function of temperature, specific leaf area and specific internode volume to estimate growth requirements at each timestep of the simulation. Biomass production is modelled using an empirical function of photosynthetically active radiation (PAR) and plant light use efficiency^7^, and any biomass that exceeds the growth requirements is converted to stored dry matter. An implementation of the ADEL-Maize model is available on the OpenAlea platform (https://github.com/openalea/adel).

**L-egume**^101^, an individual-based model (IBM), is a generic FSPM for forage legumes. The L-egume model runs simulations at a population scale and can simulate legumes growing in isolation and dense communities. The model uses L-systems to simulate a 3D geometric representation of legume architecture. The model calculates intercepted photosynthetically active radiation based on the previously described RATP model and uses Monteith’s radiation use efficiency (RUE) to calculate dry matter produced at each timestep. Dry matter allocation in the model follows an allometric relationship depending on the total biomass produced since plant emergence. Organ biomass drives N demand in the model, and soil N acquisition (using two Michaelis-Menten functions) and N fixation (at the expense of C demand) are used to meet N demand. The L-egume model is available in the OpenAlea framework (https://github.com/openalea-incubator/l-egume).

**L-Cucumber**^45^ model focuses on modelling leaf phototropism to check how it affects light interception, dry mass production and yield in a cucumber canopy. The model is implemented using L-studio combined with the CARIBU interface. The structural simulations use an extension of an original L-system model for cucumber^102^, focusing specifically on photo-morphogenic responses of canopy elements as a function of the local light environment. The model uses intercepted photosynthetically active radiation per phyllocron to calculate dry matter production per leaf. Dry matter allocation in the model is based on the distribution of sink strengths in the model, which itself is a function of the reference growth rate of each phyllocron and thermal time based leaf expansion criterion.

### Models incorporating intermediate levels of metabolic detail

#### A smaller number of multiscale models incorporate more explicit representations of selected metabolic processes while maintaining whole-plant or canopy-scale simulations

One of the most notable examples of such models is the **Arabidopsis Framework model**. **The** Arabidopsis Framework v2^62^ and its predecessor, the Arabidopsis Framework v1^61^, combine multiple independent models to form an integrated multiscale FSPM capable of being sensitive to day length and temperature in addition to environmental conditions. The multiscale model uses an FSPM^103^ to predict organogenesis and morphogenesis, and predict organ sink demand. To simulate leaf metabolism, the multiscale model uses a Carbon dynamic model^104^, which uses the FvCB model to estimate photosynthesis, convert a fixed proportion into starch, malate and fumarate, and use the remainder of photoassimilate to drive growth as sucrose. During the night, accumulated malate and fumarate are remobilised at a linear rate while a Circadian clock^105,106^ model controls starch degradation. The Circadian clock model, a photoperiodism model^107,108^, and a phytochrome signalling^108^ together drive a phenology model using photothermal time. The Arabidopsis framework models are implemented on MATLAB and available for download from the FAIRDOM hub repository (https://fairdomhub.org/investigations/123).

Another similar example is **V-Mango**^109^, an FSPM capable of simulating structural and functional aspects of mango tree development. Plant morphogenesis, during both vegetative and reproductive phases, in this model relies on building blocks called Growth Units (GUs) and follows probabilistic rules based on thermal time to simulate plant growth. This structural component in the model was implemented using a Python L-systems framework, L-Py. For metabolism, the model relies on the R-based “Cashoo” Ecophysiology model adapted for mango trees^110^ to simulate light interception, photosynthesis, maintenance costs, carbon allocation and biomass remobilisation based on structural attributes such as leaf and fruit numbers. The V-Mango FSPM also models fruit growth in cell division phases and uses thermal time-based empirical relationships to model the accumulation of organic compounds and minerals in fruits during the cell expansion phase. Integration between the functional R component and the primary Python framework was accomplished using the RPy2 module, and can be accessed through the OpenAlea project (https://github.com/fredboudon/vmango).

### Models with explicit mechanistic representation of metabolism

Only a limited number of whole-plant models currently incorporate detailed mechanistic descriptions of metabolism. **CN-Wheat**^111^ is a culm-scale FSPM with a detailed mechanistic ODE-based representation for C and N metabolism in wheat plants. The model describes above-ground parts of a wheat plant in terms of phytomer units, while the root is described as a single unit defined by its structural N content and dry mass. Leaf photosynthesis is simulated in the model by the FvCB model coupled to the Ball, Woodrow, and Berry (BWB) stomatal conductance model^99^. Photoassimilates are subsequently exported into the phloem as sucrose, stored as carbohydrates or converted to amino acids. Phloem sucrose and amino acids are used to generate carbohydrates and organic N content in the grains and roots. Additionally, in the root, the model describes nitrate uptake and C and N exudation. The model also accounts for respiration in each organ, has a cytokinin-based signalling system to regulate protein degradation, and uses a drop in tissue protein levels to trigger tissue death and nutrient remobilisation. Changes to major metabolites like sucrose, starch, proteins, amino acids, nitrates and fructans are all represented using enzyme kinetics. The CN-Wheat is implemented in Python and can be downloaded from its SourceSup repository (https://sourcesup.renater.fr/projects/cn-wheat). CN-Wheat is also available as a submodule in the OpenAlea WheatFSPM project (https://github.com/openalea/WheatFspm).

## Metabolic models can be used to study metabolism at the whole-plant level

**Fig. 2:**
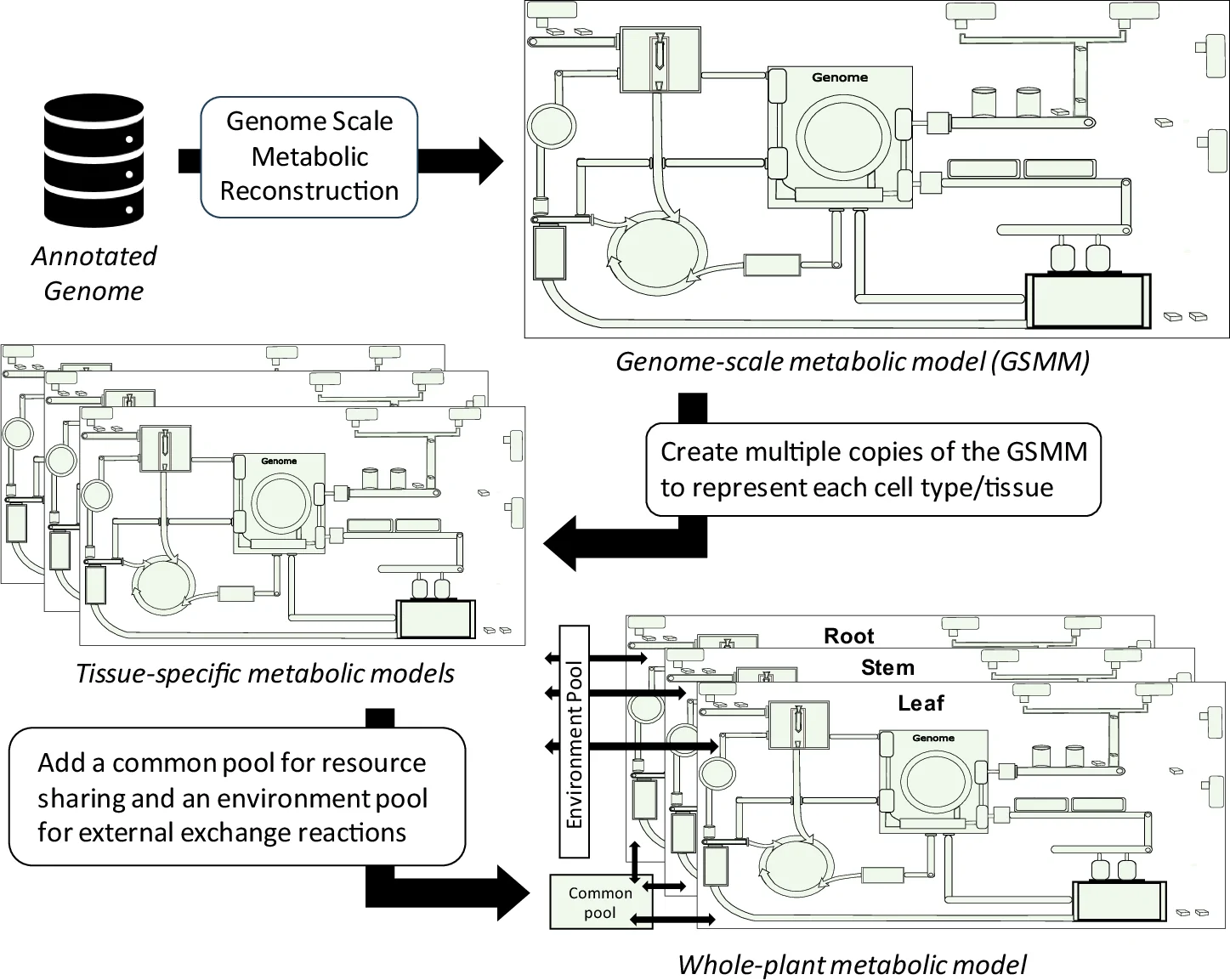
Common workflow for constructing multi-tissue metabolic models. The pipeline consists of three steps. First, curated genome-scale metabolic models are created for the organism under study. The next step involves creating multiple copies of these genome-scale models and constraining them to represent metabolism in different tissues/organs. Finally, metabolic fluxes between the tissues/organs are established by introducing a common pool, accessible to each sub-model, along with an environmental pool for access to external nutrients and energy.

Genome-scale metabolic models (GSSM) are constraint-based models used to describe the metabolic capabilities of a cell. They are stoichiometric models of metabolic and transport reactions generated based on gene-protein-reaction associations found within annotated genomes^112^. As the technology for genome sequencing, genome annotation and automated GSMM draft reconstruction has advanced and become more accessible, the number of published plant GSMM has increased^113–116^. The GSSM serves as the framework for Flux Balance Analysis (FBA) and its extensions. FBA predicts the flow of metabolites through all the reactions present in the metabolic network reconstruction based on mass balance, constraints applied, and the objective function defined. The objective function is a representation of the biologically relevant purpose of the system, and can significantly impact the modelling results. While the maximisation of the biomass^117^ is commonly used as an objective in FBA, the choice of an appropriate objective function is a persistent challenge and has been reviewed in multiple papers^118–120^. The FBA simulations provide the advantage of studying genomic or metabolic changes at the whole plant scale. Additionally, unlike the FSPMs and CSMs, they rely less on parameter fitting, so different genotypes or mutants can also be simulated. Cellular, tissue and organ-level metabolic models can be generated from GSMMs using transcriptomics data^121^ or tissue-specific constraints^122^. Such models can be scaled up to multi-tissue/ multi-organ level models. The general approach is to make multiple copies of the GSSMs based on the number of different tissues being modelled and create a common connecting link between them, generally a ‘common pool’ for resource or nutrient exchange^24,123^. The process has been described in detail in a previous review^124^ [Fig. 2].

Metabolic models have also been used to study metabolism at whole plant levels. Most notably, a multi-tissue model of Arabidopsis AraGEM model^23^ has been used to study Arabidopsis metabolism at the whole plant level. The model comprises root, stem and leaf (mesophyll) as three tissue compartments and two ‘common pools’, one each for root-shoot and stem-leaf interface. In addition to this, since the model represents diel metabolism and has distinct components representing day and night metabolism, ‘storage pool’ compartments are also included in the model to represent transitory metabolite storage. The simulations used experimentally determined growth rate and set minimum utilisation of photons as the objective function. Flux Variability Analysis (FVA) was performed on the model to understand the energy cost of transport for C and N allocation between tissues. The analysis of this model led to revelations like the fact that transport of C & N is shaped by energy-efficient strategies, and that ammonium is cheaper to transport compared to nitrate. The main advantage of the multi-tissue approach here is that it helps to understand the source-to-sink translocation of the metabolites under different constraints. However, given the fact that metabolism in the model is determined based on predefined growth rates, the model does not leave room for accounting for changes to environmental factors. The model also does not account for the remobilisation of metabolites during senescence. Additionally, the steady-state nature of models like this one also limits their scope in capturing system behaviour in dynamic growing conditions. The model also takes “a big-leaf” representation of plants, where all photosynthetic tissue is represented by a single leaf model, and as a result, does not account for the effects of plant architecture on metabolism, such as self-shading and the photosynthetic difference between sun and shade leaves.

Multi-tissue/organ metabolic models have also been used to study growth in soybean seedlings^125^. The model, which consisted of two organs (cotyledon & hypocotyl) capable of transferring nutrients between them through phloem, has been used to explore the mobilisation of reserve nutrients during the early growth period of soybean seedlings. Like the previously described AraGEM-based whole plant model, growth in this model too was constrained using experimental growth data of soybean seedlings and minimisation of fluxes was set as the objective function. As with the previous model, having been constrained based on experimentally observed growth data, the model is unable to simulate alternate growing conditions and does not account for the effect of seedling root architecture on growth^126^. Similar attempts, such as the multi-organ tomato model Virtual Young Tomato Plant (VYTOP)^127^, offer reliable predictions in multiple applications, like the role of the stem in photosynthesis, and the impact of genetic modifications. The model included a root submodel along with leaf and stem, distinct xylem and phloem, unlike the common resource pool approach, and the objective function was the minimisation of photon uptake and the sum of fluxes. The scope of the model is limited by its reliance on extensive experimental data and by the exclusion of dynamic changes and plant architecture. Along the same lines, the multi-tissue GSSM-based model of grapevine, constructed to study metabolism at various developmental stages^116^, also has the limitation of not accounting for the effects of plant architecture and dynamic changes in growth conditions during plant growth.

It has been demonstrated that some limitations of constraint-based models can be overcome by coupling them with other modelling approaches. Most notably, Bogart and Myers^128^ developed a maize leaf model by combining a GSSM with non-linear constraints associated with rubisco carboxylation/oxygenation ratio, CO_2_ leakage and phosphoenol pyruvate carboxylase (PEPC) to depict biochemical reactions capable of photosynthesis and the overall C_4_ leaves. The model also simulates tissues across a developmental gradient by creating 15 copies of a multi-tissue (bundle sheath and mesophyll) model, each representing one of the 15 different locations of a 9-day-old maize leaf and accounting for the flow of metabolites between them. Given that tissues in the different parts of the same leaf are known to be metabolically different, leaf metabolism in the study was modelled by identifying flux distributions which represented the least deviation from a high-resolution transcriptomics dataset (objective function). The model was successful in predicting that the basal portion of maize leaves acted as a sink, with the tip acting like the source. As noted by the authors, accounting for the non-linear nature of some of the metabolic processes transforms the entire model into a non-linear model. Spatially separated regions of the maize root cross-section have also been modelled using a similar approach^129^. While such approaches are able to resolve metabolic changes between different cell types in plant organs, scaling up such high-resolution models to whole-plant level spatial resolutions will require massive amounts of transcriptional data and make the process complex and computationally intensive.

Of the aforementioned, limitations due to steady state assumptions in FBA can be solved by using a dynamic FBA (dFBA) model^130–132^. In dFBA, the total time period for modelling is split into several individual time steps (static optimisation) or the entire time period solved simultaneously (dynamic optimisation)^133^ to try and capture the dynamics of the system. The approach has been extensively reviewed in Kleessen and Nikoloski^134^. At the whole-plant level, dFBA has been used to study biomass partitioning between root and leaf during growth in Arabidopsis plants^135^. The model contains distinct representations of day and night metabolism in both root and shoot, with a common pool allowing the transfer of metabolites between the different organs, much like the previously described multi-tissue AraGEM-based model^23^. N uptake, sucrose transport, and amino acid transport were all constrained to simulate day and night conditions and the model was solved in a day step based on the quasi-steady state assumption with fluxes optimised in accordance with the balanced plant growth^136,137^ based on dynamic relative organ growth rate constraints (objective function). The model is capable of providing a detailed flux map for C and N metabolism in Arabidopsis during the various developmental stages, as well as different resource-limiting growing conditions. Additionally, the model is also sensitive to root and shoot loss due to external influences like herbivory in limited and optimum N availability. Nevertheless, the model has many limitations. While N uptake in the model was accounted for based on enzyme kinetics, other core processes like photosynthesis are modelled based on incident light and total leaf area alone. Since the focus is on biomass partitioning, other physiological functions are not well-detailed in the model.

The quasi-steady state assumption in the static optimisation-based approach (SOA) dFBA offers reduced complexity while modelling the system compared to the dynamic optimisation-based approach dFBA^134^. However, as in the Shaw and Cheung model^135^, it reduces the temporal resolution of the model, providing a single flux value for a whole day (or the time period chosen for optimisation). Instead of optimising the whole system (including the day and night temporal phases) at once, other methods like Optimisation and Explicit Runge-Kutta based approach (ORKA) dFBA^138^, offer a more practical solution to increase the temporal resolution by optimising the system for maximal growth in hourly time points. The study used metabolic models of four tissues (stem, root, seed, leaf) to assemble a whole plant Arabidopsis model, and simulated plant metabolism from germination to senescence in hourly timepoints. A total of around 1464 time points were modelled. In the ORKA framework applied, each of these time steps is again optimised in three subtime points. At first, the algorithm solves the system by standard FBA and updates the growth rate and metabolite concentrations, and then for each of the subtime points, it re-estimates all values by the third-order Runge-Kutta method (Heun’s Third Order Rule) and solves each step, finally taking a weighted average to get the growth rate and biomass. The algorithm also uses a trapezoid rule-based integral approximation to estimate the metabolite concentration at each time step. Additionally, the model accounts for biomass allocation, tissue/organ-level maintenance costs, and senescence. Given these enhancements, the model was also able to describe the flow of water and sulphur metabolism across developmental stages in detail. Although this approach has improved resolution, it is computationally intensive and mathematically complex.

Overall, a wide range of approaches can be applied to study metabolism in plants at the whole plant level. However, in general, there are certain limitations to using constraint-based models alone. Firstly, these models do not account for the architectural details of the plant and instead use simplified representations, such as total leaf area, to account for plant structural changes. Steady state assumptions used by popular constraint-based modelling algorithms also limit the ability of the models to account for dynamic changes in environmental conditions and plant development. Dynamic FBA approaches, which offer solutions to this problem, are computationally intensive and mathematically complex. Inclusion of precise enzyme kinetics also faces the same challenges, in addition to the requirement for more data. Previously described examples imply that although modelling aimed at solving any one or two issues seems possible, solving multiple issues and simulating development at different scales using constraint-based modelling alone is not possible. Hybrid models, which combine different classes of models, could be a better choice for handling these issues. Models which combine constraint-based models with an FPM, FSPM or CSM could prove to be effective in describing plant metabolism and development at various scales, offering a greater scope for insight into plant growth.

## Unidirectional integration of the metabolic model with other models to improve the representation of metabolism

**Fig. 3:**
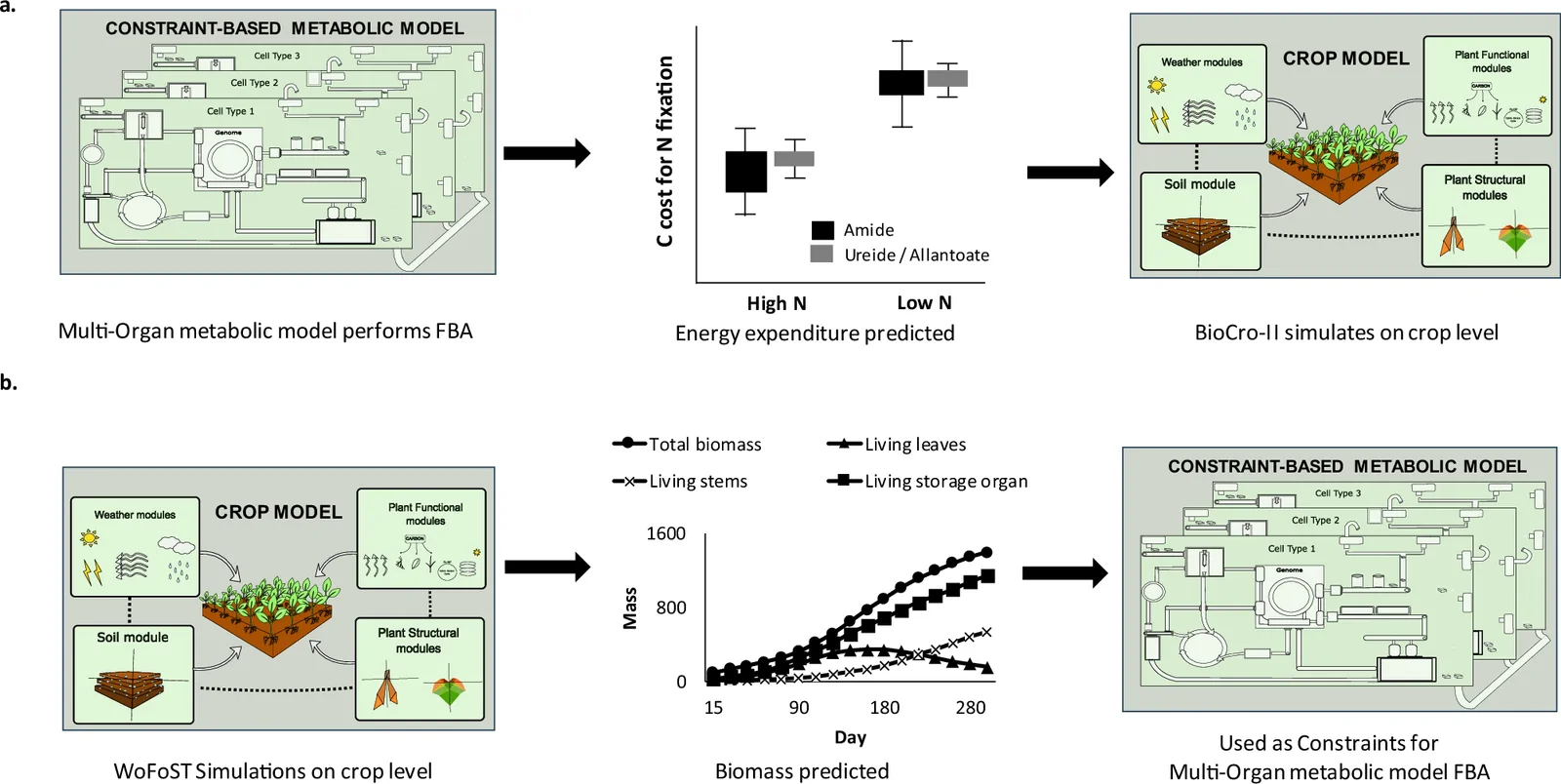
Two types of unidirectional integration have been applied in current hybrid models involving constraint-based models. **a** A soybean constraint-based model has been used to predict the C cost required to support N fixation under abundant and low N conditions, and this cost was used to improve yield estimations in the Soybean BioCro model. **b** A crop model (WOFOST) was used to predict the daily change in plant organ mass in rice cultivation. These values were passed on to a constraint-based whole-plant rice model to enable the model to predict metabolic fluxes within each organ. In both cases, only unidirectional communication between individual models is achieved.

Hybrid modelling, which involves the integration of two or more different modelling approaches, has been shown to be capable of combining the strengths of the individual models while overcoming their individual limitations. In general, hybrid modelling can be either unidirectional or bidirectional based on how communication between the models is designed. In unidirectional integration, modelling results from one model are passed as input values to another model and complete a single run of the whole simulation. However, the second model does not communicate back to the first model, effectively a one-way communication (Fig. 3). One of the best examples for this type of integration is the one observed when Soybean-Biocro, a soybean variant of the semi-mechanistic dynamic crop model BioCro^55^, was integrated with the GSMM-derived nodulated whole-plant metabolic model^139^. The Soybean-BioCro model simulates hourly growth rates of leaf, stem and pod using mechanistic modules (such as the classic FvCB model for photosynthesis) and non-mechanistic modules (such as the SOYDEV soybean developmental stage model), but is unable to account for N-fixing nodule formation commonly found in soybean^140^. Integration of the metabolic model, a three-organ constraint-based model containing the shoot, root and nodule with a bacteroid model inside, aims to cover this limitation of Soybean BioCro. The metabolic model calculates the minimal metabolic costs associated with N-fixation under N-rich and N-limited soil conditions and uses it to correct whole plant biomass in the Soybean Biocro model. This integration of a metabolic model with a crop model predicts that the nodulating soybean has up to a 27% reduction in its yield based on the C allocation.

Alternatively, instead of the FBA model passing on its result to the crop model, a more popular configuration is for the crop model to run first and pass on the results as constraints to the FBA model (Fig. 3). One of the best examples of such an integration is the Shaw and Cheung rice model^141^. In this hybrid model, the WOFOST rice CSM is used to constrain organ biomass accumulation/remobilisation rates in a GSMM-derived multi-tissue rice metabolic model (similar in architecture to Shaw and Cheung 2018^135^ described earlier), enabling it to model metabolic fluxes under water-stressed conditions. The WOFOST crop model is a collection of multiple modules interacting together to simulate crop-level growth^47^. Temperature-driven phenological development modelling is fundamental to plant growth in this model. The model uses absorbed radiation and photosynthesis-light response curve data to determine the daily gross CO_2_-assimilation rate, and temperature to drive phenological development. It also accounts for growth and non-growth (maintenance) associated respiration costs. Transpiration rate in this model depends upon the leaf area, evaporative demand and CO_2_ levels. Biomass partitioning among different organs in the model relied on predefined values from biomass allocation partitioning tables. WOFOST also accounts for some plant architecture traits, such as leaf area defined by the Leaf Area Index (LAI). LAI is determined by temperature in the initial growth stages and biomass accumulation rate in the later stages. The model also takes into consideration leaf senescence by accounting for physiological ageing, self-shading, and water availability. While it does not account for stem height, a simplified root architecture based on initial and maximum root depth is accounted for in the model. In their hybrid modelling architecture, Shaw and Cheung constrained a constraint-based whole plant model using WOFOST-predicted leaf, stem and seed growth rate, thereby overcoming multiple limitations of constraint-based models. Thanks to the dynamic nature of the WOFOST model, the integration is able to overcome the steady state limitation of constraint-based models and simulate dynamic growth. Leaf architectural details and their influence on biomass assimilation have also been accounted for in this integration. However, the hybrid model had its limitations. Although quite descriptive when it comes to crop models, WOFOST does not simulate changes in root biomass and therefore the predicted metabolic flux distributions did not include root metabolism. Additionally, here the constraint-based model only provides a representation of potential flux distribution and does not contribute to growth estimations made by WOFOST. As a result, although the hybrid model can study changes in flux distributions and predict gene/protein expression for scenarios that WOFOST can model, any metabolic changes to which WOFOST is insensitive cannot be studied using this model.

Another example of hybrid modelling, which follows a similar design, is the barley multiscale whole-plant model^142^. This hybrid model involves the integration of a constraint-based whole plant model with an FPM, the ProNet-CN. ProNET-CN is capable of modelling photosynthesis, primary C metabolism and leaf N status using Michaelis-Menten and concentration gradient-driven kinetics^143^. The hybrid model uses SOA dFBA to simulate barley development at the whole plant level. The constraint-based component of the hybrid model is a multi-organ metabolic model reconstructed using curated models of stem, leaf, seed and a simplified root model. dFBA flux constraints were added to the metabolic model based on major C and N fluxes in ProNet-CN, and fluxes were optimised for the minimisation of C uptake and the sum of all fluxes (objective function). This setup allowed the model to estimate average daily fluxes across multiple leaves, stem and ear in Barley plants from 53 to 70 days after sowing. The model primarily focuses on C metabolism and uses a fixed C-N ratio to model N metabolism indirectly. The hybrid model, through ProNet-CN, uses a thermal time concept to account for plant development (leaf emergence and organ initiation). While the resulting model is able to account for senescence and can capture the transition of Barley stems from sink to source, it has limited representation of N and root fluxes, given the fact that the root model is represented by only three exchange reactions. The primary focus of the ProNet-CN is to capture biochemical mechanisms in the whole plant, and as such, the hybrid model has little representation for plant architecture or structural details.

Compared to CSMs, FSPMs are better at accounting for individual variability in plants^144^. However, hybrid models where FSPMs are coupled with constraint-based models are yet to be explored in depth. As mentioned earlier, the Barley breeder model^42^ does combine growth-associated processes acting across multiple hierarchical levels to account for Gibberellic acid signal transduction and shade avoidance on plant growth. The model, however, does so using only rule-based logic to calculate growth. When it comes to FSPM constraint-based model integrations, the maize lateral root hybrid model by Hunt et. al., 2023^145^ is also notable because of its integration of a root anatomy FSPM RootSlice^146^ with a multi-tissue FBA model. The FBA model, here, relies on RootSlice to predict changes in tissue-specific cell volumes and subcellular organelle volumes during root elongation, thereby enabling the FBA model to identify a minimal flux distribution that represents metabolism during root growth (objective function). However, in addition to the fact that the hybrid model only represents root metabolism, this is also a unidirectional integration where the FBA model is influenced by the RootSlice model but not the other way around. Still, the integration allows the FBA model to model tissue-specific metabolic fluxes and predict aerobic fermentation in cortical, endodermis and pericycle cells.

## Bidirectional integration of models during hybrid modelling

In contrast to the unidirectional integration discussed earlier, a bidirectional integration framework allows the second model to communicate back to the first model. Such integrations involving constraint-based metabolic models are rare. A notable example of such a design is the hybrid e-Photosynthesis-FBA model. e-Photosynthesis is a kinetic model (ODE-based) of photosynthesis, primarily focused on light reactions and C metabolism^16^. Due to the large number of kinetic parameters and experimental data required to establish processes in the model, the model lacks many important components of leaf primary metabolism, including amino acid biosynthesis and lipid metabolism. To overcome this problem of limited metabolic coverage, the e-Photosynthesis was integrated with a constraint-based leaf metabolic model with an objective function to maximise phloem export rate (in mature leaves or biomass accumulation rate in the case of a growing leaf)^147^. The study also demonstrated the impacts of the two types of integration approaches: a Loosely Coupled (LC) unidirectional integration and a Tightly Coupled (TC) bidirectional integration. In the LC hybrid model, the e-photosynthesis model predicts Photosystem II (PSII) electron flux and Rubisco carboxylation to oxygenation (Vc/Vo) ratio based on a given atmospheric CO_2_ concentration and light intensity, and passes them on as constraints for the corresponding reactions in a constraint-based leaf model for FBA, similar to the dFBA frameworks described earlier. In the TC hybrid model, e-Photosynthesis fluxes associated with starch synthesis and plastidic efflux/influx of glyceraldehyde 3-phosphate, glycerate and glycollate were used to model metabolism in mature and growing leaves using the constraint-based model. The modelling architecture then enabled the constraint-based model to pass on any additional chloroplastic ATP and NADPH costs (encountered during amino acid and lipid synthesis, processes absent in the e-Photosynthesis model) back to the e-Photosynthesis model, forcing the e-Photosynthesis model to re-run. The process is repeated until both models converge onto a single flux distribution. While both LC and TC configurations enhance the metabolic coverage of the model, the unidirectional LC model was found to significantly underestimate starch accumulation in field-grown soybeans under both ambient and elevated atmospheric CO_2._ These results suggest that the bidirectional TC integration helps preserve the prediction accuracy of the more accurate e-Photosynthesis model while improving its metabolic coverage using the constraint-based model. Similar bidirectional integrations are yet to be realised at the whole plant level.

## Limitations and future prospects

Although integration of the metabolic models with FPMs, FSPMs, or crop models has been shown to enhance their mechanistic nature and thereby the interpretation of results, certain limitations exist in the current implementations. The primary advantage of a hybrid model is that, when done correctly, it can help to overcome the individual limitations of each model, be it by introducing sensitivity to nutrient limitations or overcoming the issue of limited metabolic coverage. While most current implementations accomplish this, they rely primarily on one-way (or unidirectional) integrations wherein one of the models runs completely independently while the other is influenced by the first model. For example, in the rice WOFOST + FBA hybrid model^141^, the WOFOST model provides growth rates which constrain the FBA model, but the FBA model has no way of influencing the WOFOST results. As a result, the resulting hybrid model will be unable to account for the effects of any metabolic engineering (such as the introduction of a photorespiratory bypass) on plant growth. Ideally, the individual models should be able to give appropriate feedback to each other. Apart from the e-photosynthesis+FBA model^147^, all the other previously described examples of hybrid models are devoid of such two-way feedback (bidirectional integration) mechanisms. However, it should be noted that the e-photosynthesis models only consider leaf-level metabolism, and yet each bidirectional TC run was shown to take at least 156 seconds (assuming the e-photosynthesis and FBA models take 35 and 2 seconds per instance, respectively, and four instances are required for the models to converge). Model execution time in the case of a unidirectional LC run would only be 37 s, less than one-third the execution time of the bidirectional TC configuration. Similar implementations at the whole plant level, while ideal, would be computationally intensive. Such challenges can, however, be overcome in the future thanks to advances in computational power and optimisation strategies. To this end, recently, a C + + variant of the e-photosynthesis model was shown to have significantly reduced the model runtime from 35 seconds using the MATLAB ode15s solver to under 1 second using the C + + CVODE solver^148^.

Another limitation of many existing whole-plant hybrid models is the limited metabolic coverage of these models. As they should, metabolic models used in hybrid models focus on the organs/tissues relevant to the objective of the study. For example, in the N_2_-fixing soybean FBA+BioCro model^139^, the metabolic model represents only a shoot, root and bacteroid-containing nodule, tissue abstractions relevant for the specific objective of determining C cost associated with N_2_ fixation by the nodule. This representation of whole plant metabolism will be found to be lacking in the case of studies involving the metabolism of senescing leaves and/or developing seeds. Similarly, in the case of the rice WOFOST + FBA model, given that WOFOST cannot predict root growth rate, the FBA-based metabolic model contains only representations of seed, leaf, and stem, and none for the root. The lack of representation of any organ can lead to miscalculations in source-sink dynamics. Given the lack of a root, all the nutrients in the WOFOST + FBA rice model are obtained directly from a ‘common pool’. The energy demand for these uptakes and other related processes in mature roots is not accounted for by the model. Similarly, due to the lack of representation of root growth demands, the photosynthetic and N-assimilation demands on the source tissue are underrepresented. As a result, the model, as is, may not be suitable for studies involving the study of source leaf and root metabolism. An improved whole-plant representation, by including a root model and estimating root growth based on allometric scaling^149^, may be necessary for a comprehensive analysis of biomass allocation and studying source-sink interactions.

A fundamental assumption in FBA and other constraint-based models is that the metabolic system is at steady-state. Capturing dynamics in plant growth and metabolism, hence, requires dividing simulation time into smaller intervals and assuming the system is at pseudo-steady-state during the time, as in static optimisation dFBA. In the case of a plant’s growth cycle, such a strategy would result in a large number of time steps, reliance on more temporal data and a higher computational load. As a result, simulating the entire life cycle of species with a higher life span will be time-consuming. However, as previously stated, thanks to improvements in computational power and optimisation algorithms, these issues may become trivial in the near future.

Current whole plant integrations also have limited representation of plant structure. Models like the ProNet-CN lack representation for plant architecture and cannot account for plant canopy effects like self-shading. In WOFOST, when leaves attain a critical leaf area, self-shading contributes to leaf death. In this manner, the model accounts for the limited effects of plant structure. However, in WOFOST, the model is incapable of predicting root growth, and it may underestimate plant growth demands. Similarly, the Soybean BioCro model also describes only the functional processes associated with the plant root and not any structural details. A better representation of plant structure can improve predictions from associated functional modules. In this context, FSPMs that predict better structural details offer better candidates when constructing mechanistic hybrid models.

**Fig. 4:**
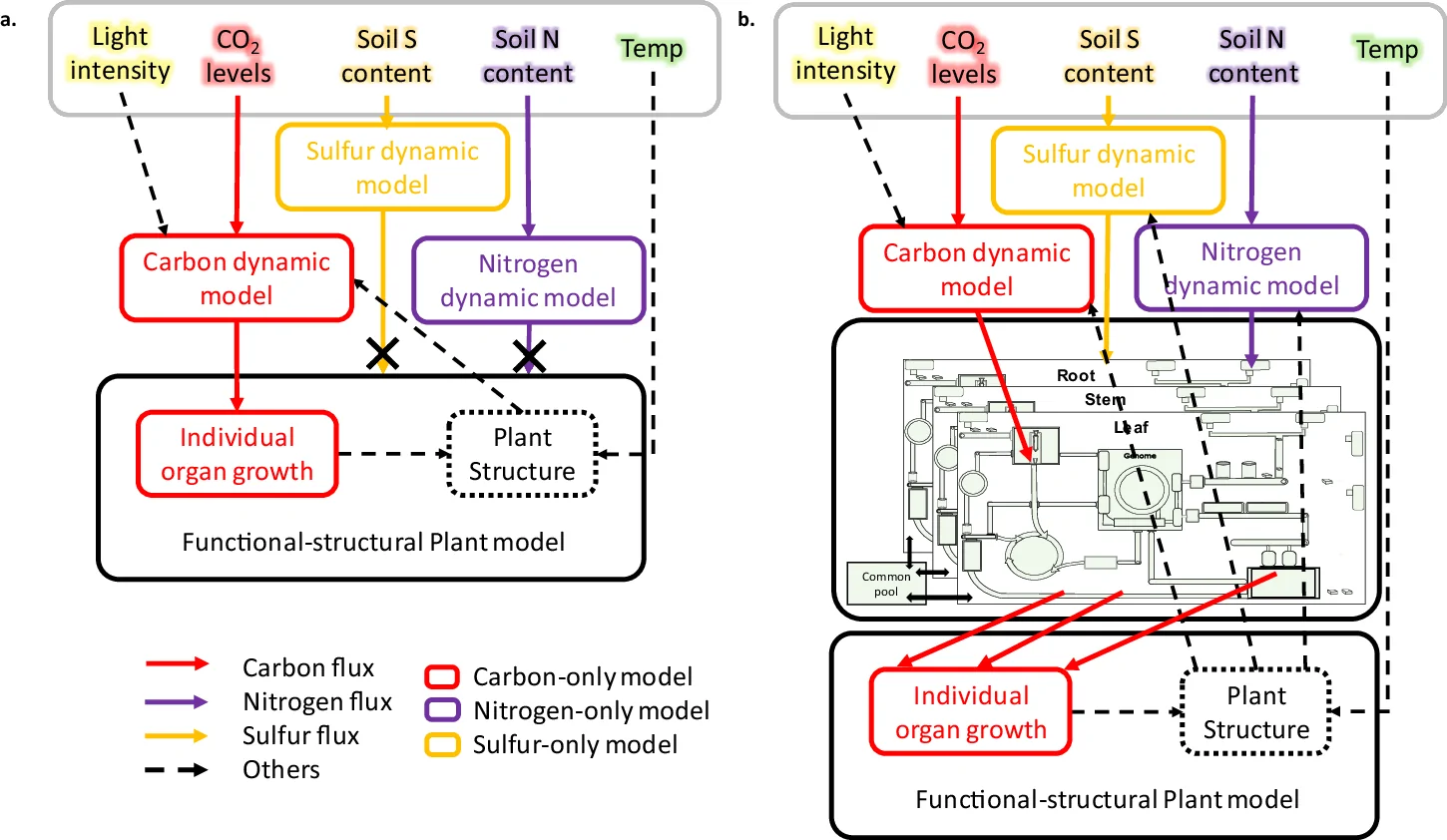
A schematic representation highlighting the advantage of integrating constraint-based models into FSPMs. **a** Similar to the Arabidopsis framework model v2, many FSPMs only account for carbon to represent biomass, and as a result, these models are insensitive to the availability of other important nutrients. Integrating a dynamic nitrogen or sulphur model to such FSPMs requires extensive restructuring of the FSPM architecture. **b** Constraint-based models are perfect as a bridging layer for connecting modules associated with different nutrient acquisitions. Since constraint-based models can have all metabolic pathways, metabolic fluxes from all modules can be integrated into it in the form of constraints. Through constraint-based models, all these models can influence plant growth and architecture in FSPMs without needing additional restructuring. The FSPMs can also feed back into the modules, enabling bidirectional communication and convergence between modules.

One of the best examples of integrating multiple models together so as to improve the predictive power of the individual models is the Arabidopsis Framework model^61^. As described earlier, the FMv1 combines a C Dynamic Model (CDM), an FSPM, a photothermal model (PTM) and a photoperiodism model (PPM); and is capable of simulating Arabidopsis plant growth until flowering. The updated version of the framework model, FMv2^62^, has an improved representation of starch and malate metabolism. However, like many current FSPMs, the model has no representation for N, P or S, and as such is unable to capture the response to the limited availability of any of these nutrients. Additionally, given the lack of any representation of non-C biomass components in the FSPM framework of the FMv2 model, the multiscale model is unable to incorporate modules representing non-C assimilation and metabolism without significant restructuring. An ideal workaround for this would be to use a constraint-based metabolic model to integrate metabolism in the model [Fig. 4]. A constraint-based metabolic model can incorporate inputs from various models, be it a kinetic model of metabolic processes or a dynamic model of gene expression, as constraints and use steady-state assumptions to solve for the remainder of the metabolic system. Information on organ mass and microenvironmental conditions from FSPMs can be used to constrain and operate the aforementioned hybrid metabolic model, and the resulting integrated growth rate from the constraint-based model can be passed on to the FSPM [Fig. 4]. In such a two-way integrated hybrid multiscale modelling framework, individual models can continuously communicate with each other to drive the plant growth.

## Conclusion

Mathematical modelling offers a great advantage in understanding plant growth. At the whole plant-level modelling metabolism requires a multiscale approach. Current whole plant modelling approaches have yet to achieve this, as they mostly rely on empirical modelling. Constraint-based metabolic models offer an alternative to empirical modelling of metabolism and reduce the reliance on parameterisation. However, using constraint-based models for growth at whole plant levels faces multiple challenges, including limited representation of structural and functional mutual dependencies, the limited scope for accounting for dynamic changes and the inability to incorporate non-linear responses to environmental parameters. Although methods like dFBA attempt to solve some of these issues, using constraint-based models alone has limited potential in solving them simultaneously. Hybrid models, which combine a constraint-based metabolic model with other model types such as kinetic models, FPM, FSPM, and CSM, offer a better platform for the dynamic representation of metabolism and connecting it with plant structure. Although modelling at multiple scales can be achieved through this approach, to date, the number of attempts is very small. Most of the current approaches are limited to one-way communications between models; this does not utilise the full potential of the constraint-based models or the hybrid models. The methods of integration of these models are also unique for each, with no general approach. Hybrid modelling with feedback mechanisms between metabolic models and the other components has the potential to substantially improve plant growth simulations and analysis. Constraint-based metabolic models have the potential to act as a platform for integrating multiple models and simulating bidirectional communications between models. However, ensuring numerical convergence and physiological consistency between models remains a major challenge in such multiscale hybrid frameworks, particularly when growth, sink strength and assimilate partitioning dynamically influence one another. Efforts in these aspects would accelerate the continuous adoption of models describing the various plant processes into multiscale models capable of facilitating research into future-proofing crop yield.

## Data Availability

No datasets were generated or analysed during the current study.
